# Probiotic Properties of Lyophilized Cell Free Extract of *Lactobacillus casei*

**DOI:** 10.17795/jjnpp-8564

**Published:** 2013-07-23

**Authors:** Afrooz Saadatzadeh, Mohamma Reza Fazeli, Hossein Jamalifar, Rassoul Dinarvand

**Affiliations:** 1 Department of Pharmaceutics, Faculty of Pharmacy, Ahvaz Jundishapur University of Medical Sciences, Ahvaz, IR Iran; 2 Department of Food and Drug Control, Faculty of Pharmacy, Tehran University of Medical Sciences, Tehran, IR Iran; 3 Nanotechnology Research Centre, Faculty of Pharmacy, Tehran University of Medical Sciences, Tehran, IR Iran

**Keywords:** Freeze Drying, Lactobacillus casei, Probiotics

## Abstract

**Background:**

In recent years there have been considerable interests in the use of probiotic live cells for nutritional and therapeutic purposes. This strategy can be concomitant with some limitations such as survival of live cell during the GI-transit and their effective delivery to target tissues upon ingestion. Several attempts have been made to overcome these limitations such as their microencapsulation, spray-drying and lyophilization.

**Objectives:**

In this study extract of cultured probiotics without cells was evaluated for its antimicrobial effects, antioxidant activity, and its stability.

**Materials and Methods:**

In this work the potential of lyophilized-cell-free-probiotic-extract (LPE) as a suitable alternative strategy for the preparation of probiotic-products was investigated. The main aim of this study was to find out the antibacterial and antioxidant activity of LPE and also its stability. LPE was obtained by centrifugation and subsequent lyophilization of the collected supernatant from culture media of *Lactobacillus casei*. An enzymatic reagent-kit was used for detection of its content of lactic acid. Antibacterial test was performed using agar cup-plat-method, the DPPH scavenging -assay was used to determine its antioxidant activity and during a storage course, LPE was under a long-term stability study.

**Results:**

Results showed that, LPE had more antipathogenic effects, antioxidant activity, and stability during storage-time when compared to fresh probiotic-extract.

**Conclusions:**

Employing the LPE as a new approach, gives novel concept of probiotic-products in food and medical marketing.

## 1. Background

Probiotics, or friendly bacteria, are normal resident flora of intestinal tract, which can be formulated into many different types of products. The term “probiotic” was first proposed in 1965 by Lilly and Stillwell as “microbial derived factors promoting the growth of other microorganisms” (1). In 1989, Fuller defined a probiotic as “a live microbial dietary supplement which has positive effects on the host by improving its intestinal microbial balance” (2). Although numerous definitions have been proposed since then, none has been completely satisfactory. According to FAO and WHO explanation, probiotics are defined as living microorganisms, which upon consuming in adequate amounts can exert their profit health effects (3, 4). Species of *Lactobacillus* and Bifidobacterium are the most commonly used probiotics, while other microorganisms such as *Saccharomyces cerevisiae* and Escherichia coli strain nissle have also been used as probiotics (5-7). Lactic acid bacteria is one of the most commonly used probiotics (8, 9), which has potential for production of metabolites including; organic acids, bacteriocins, enzymes, vitamins, and other unknown metabolites (10). Probiotics have a variety of beneficial effects such as; prophylaxis of intestinal infection in livestock animals, prevention of atopic dermatitis, lactose intolerance, gastrointestinal infections, and various types of diarrhea as well as Inflammatory Bowel Disease (IBD) and Irritable Bowel Syndrome (IBS), eradication of Helicobacter pylori liver diseases, urogenital infections, and GI disorders in human (5, 7). Briefly, probiotics bacteria exert their beneficial effects via two mechanisms; i) Direct effects of live cells, and ii) Indirect effects via producing wide variety of metabolites or biogenics (6). Several attempts have demonstrated the ability of *Lactobacillus* strains to inhibit pathogenic microorganisms related to the production of lactic acid, other organic acids and bacteriocins (11-18). While probiotics are considered as safe and harmless microorganisms, they have several limitations which can affect their consumption route such as oral administration of bacterial cells (19). Besides, ingestion of live microbial cells in immunocompromised patients may be associated with risk of serious infections as administration of *L. rhamnosus* may result in liver abscess (20). Many attempts have been made to find solutions to overcome these problems. For instance, applying different microencapsulation strategies for live cells (19, 21-23), various dried viable cells (24), and indirect feeding of microflora metabolites to the animals (10).

## 2. Objectives

The use of cell-free probiotics extract can be considered as an alternative method. For this purpose, in this study cultured probiotics extract without cells was evaluated for its antimicrobial effects, antioxidant activity, and its stability. Furthermore, Lyophilization as a good method for enhancement of its properties was studied. 

## 3. Materials and Methods

### 3.1. Microorganisms and Culture Media

The probiotic strain was *Lactobacillus casei* ATCC 39392, while the target pathogenic bacteria included *Staphylococcus aureus* ATCC: 6538, *Pseudomonas aeruginosa* ATCC: 9027, *Escherichia coli* PTCC: 1399, Salmonella typhimurium ATCC: 14028 which were all obtained from the stock cultures of the Department of Drug and Food Control, Faculty of Pharmacy, Tehran University of Medical Sciences, Tehran, Iran. The culture media used for propagation as well as identification of bacteria included; de man rogosa Sharpe (MRS) broth and agar, triple sugar iron-agar (TSI), sulfide hydrogen-indole-motility (SIM), and muller-hinton agar (MH-A) Lactic acid with 99% purity, all from Merck, Germany, The enzymatic lactate reagent kit was obtained from Chem-Enzyme Co., Tehran, Iran.

### 3.2. Microbial Culture

The bacteria were cultured in MRS Broth medium at 37˚C for 24 hours and maintained on MRS agar, anaerobically. Anaerobic conditions were achieved using an anaerobic glove box (Anoximat incubator, Germany) with 95% N_2_, 5% H_2_, 6% O_2_, and 5% CO_2_. to check the colonies purity, Gram stain, culturing in both medium; TSI agar and SIM were used. For the control of *Lactobacillus* strain, the fermentation test of the carbohydrates was performed. MRS broth medium was prepared without sugar, and each of carbohydrates was added into separated medium in every tube. The suspension of microorganism (MO) was inoculated into these tubes, and was incubated in 37˚C for 4 days.

### 3.3. Growth Rate Determination of Probiotics on Culture Media

For estimation of the growth rate of *Lactobacillus casei*, 1 mL (equal to 102 cfu/mL) of the fresh cultured *Lactobacillus* was inoculated to 100 mL of MRS broth (as the standard laboratory medium). After mixing, flask was incubated at 37˚C for 24 hours, and viable cells count were examined by pour-plate method at predetermined time intervals.

### 3.4. Antipathogenic Effect of *Lactobacillus casei*

Fresh culture of *Lactobacillus casei* was cultured in MRS-broth, and incubated in anaerobic condition at 37˚C for 24 hours until the cell densities reached 108 CFU/mL. For spot method, 10 µL of this medium (fresh inoculum) was spotted on the center of a plate containing MRS-agar and incubated in anaerobic condition in 37˚C for 24 hours. afterward, 1 mL of an overnight culture of each pathogens (*Staphylococcus aureus*, *Pseudomonas aeruginosa*, *Salmonella typhimurium*, *Escherichia coli* and MRSA (methicillin resistant *Staphylococcus aureus*) suspension (0.5 McFarland) was mixed with 10 mL of semisolid MH-agar, and cultured on the surface of spot plate as a second layer. The plates were incubated at 37˚C for 48 hours, and the colonies examined for formation of inhibition zones. The pathogenic strains without *Lactobacillus* were considered as negative control and the *Lactobacillus* without pathogenic bacteria was considered as positive control. The experiment was repeated in triplicate.

### 3.5. Cell-free Culture Supernatant Preparation

The Lactobacillus strain was grown anaerobically in 100 mL of MRS broth for 24 hours at 37˚C. Supernatant was obtained by centrifuging the medium at 4000 rpm for 15 min at 20˚C. Centrifuged supernatant was passed through a sterile 0.22 µ-pore-size filter unit (Millex GS Millipore). The filtrate was collected as the mixture of metabolites and then was kept at 4˚C.

### 3.6. Etiology of Antibacterial Activity of Cell-free Supernatant

To determine whether the antimicrobial activity of *L. casei* recorded, was strongly related to production of lactic acid, the “Agar Cup-plate method” was used. 20 mL of sterile MH-agar medium was poured into sterile petri-dishes and allowed to solidify. The petri-dishes were incubated at 37˚C for 24 hours to check the sterility. The pathogenic strains were adjusted to108 cfu/mL by adding sterile water and spread on the surface of MH-agar. Four bores were made on the medium in each plate using sterile borer. As the control for Bacteriocin efficiency, the pH value of supernatant was adjusted to 7.0 by addition of 0.1 N NaOH and 100 µL of fresh supernatant and neutralized one were added to the respective bores. 100 µL of lactic acid and H_2_O_2_ at a concentration of 3% v/v were taken as standards. The plates were kept at 4˚C for 30 minutes for diffusion and then, incubated at 37˚C for 24-48 hours with appropriate positive and negative controls, and zone of inhibition were observed and measured.

### 3.7. Lactic Acid Assay

Quantitative detection of lactic acid in acquired supernatant was performed using an enzymatic lactate reagent kit (chem. Enzyme). This assay method was based on two stage oxidative reactions as follows;

**1):**
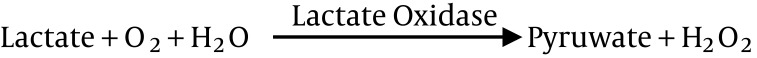


**2):**
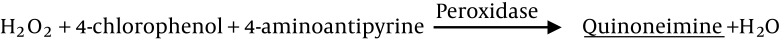


The absorbance of Quinoneimine as the final colored product was measured at 500 nm wavelength (UV detector, S-3100, Scinco, Korea). Lactic acid level of supernatant was expressed as mg/dL. Standard curve was prepared using Lactic acid with 99% of purity as the reference standard in different concentrations. The assay test was repeated in triplicate.

### 3.8. Comparison of the Content of Lactic Acid Between Obtained Supernatants From Different Times of Culture

To investigate the effect of culturing time on production of metabolites especially lactic acid, *Lactobacillus casei* with the same concentration was separately inoculated to three flasks containing 100 mL of sterile MRS broth medium. After different incubation times: 24, 48, and 72 hours at 37˚C, all the cell–free supernatants were collected separately and were analyzed for assay of lactic acid. Besides, fresh extracted supernatant was lyophilized for 48 hours in -50˚C by Freeze-dryer (Lyotrap Plus, LTE Scientific Ltd, UK) to obtain a concentrated supernatant, and its content of lactic acid was also determined.

### 3.9. Long Term Stability Study

The acquired supernatant was stored at 4˚C for 9 months, and its lactic acid concentration was assayed at certain intervals. The value of pH was also determined using a pH meter (Met Rohm®) during storage course and results were recorded.

### 3.10. Antipathogenic Effect of Supernatant

Agar Cup-plate method as described previously, was used to detect antipathogenic effects of acquired supernatant during its storage course and post lyophilization. Five bores were made on the pathogenic seeded medium in each petri-dish, and respectively poured by 100 µL of fresh supernatant on 0 day (Sup-0), 30 days post extraction (Sup-30), 60 days post extraction (Sup-60), and 90 days post extraction (Sup-90), and Lyophilized Probiotic extract (LPE). After keeping at 4˚C for diffusion, the plates were incubated for 48 hours at 37˚C, and zone of inhibition was observed and measured.

### 3.11. Determination of MIC of Both Probiotic Extract Against Pathogen Strains

The conventional macrodilution tube method was used to determine the minimum inhibitory concentration (MIC) of both probiotic extracts; fresh supernatant (Sup-0) and LPE with respect to five pathogenic strains. A stock solution of Sup-0 was prepared in sterile Muller-Hinton broth (64 mg/mL) which was further diluted in MH-b to reach concentration range of 4 mg/mL to 64 mg/mL. LPE was also dissolved in MH-b to reach a concentration range of 0.031 mg/mL to 0.5 mg/mL. Afterwards, 100 µL culture of one of the test bacteria, grown to the early stationary growth phase in MH-b, was added to 1 mL of MH-b in tube as final concentration of bacteria in individual tubes was adjusted to about 5 × 10^6^ CFU/mL. Control tubes contained; only culture media without any antibacterial agent, culture media with LPE, culture media with pathogenic strains (5 × 10^6^ CFU/mL), and culture media with Sup-0. After 24 and 48 hours incubation at 37˚C, the test sample was determined as lowest concentration that could inhibit visible bacterial growth for 24 hours (25, 26).

### 3.12. Antioxidant Effect of Supernatant

The Antioxidant activity of supernatant was determined using the stable free radical 2, 2-diphenyl-1-Picrylhydrazyl (DPPH) scavenging assay. DPPH solution was prepared in a methanol/water (50:50) solution. 1 mL of samples (LPE, Sup-0, Sup-30, Sup-60 and Sup-90) was added to 5 mL of DPPH solution and after 3 hours of reaction at 37˚C in a vessel mounted on a shaker in incubator, the absorbance was measured at 517 nm. Ascorbic acid solution (1 M) was used as the reference standard, Lactic acid solution (1 M) was considered as a positive control, DPPH solution without sample was used as control and methanol/water solution (50:50) was as blank. Duplicate measurements were performed, and their scavenging effect was calculated based on the percentage of DPPH scavenged using the following equation:

Antioxidant Activity, % = DPPH radical scavenging, % = [1 - (As/Ac)] × 100

Here, Ac = absorbance of control which is 0.817 for DPPH and As = absorbance of sample solution (27, 28).

### 3.13. Statistical Analysis

Results are expressed as mean values ± standard error of the mean (SEM). The data was analyzed by one-way ANOVA followed by Turkey’s post hoc test for multiple comparisons to ensure the variances of data normal distribution. P-value less than 0.05 was considered significant.

## 4. Results

### 4.1. Growth Kinetic of L. casei

**Figure 1.**
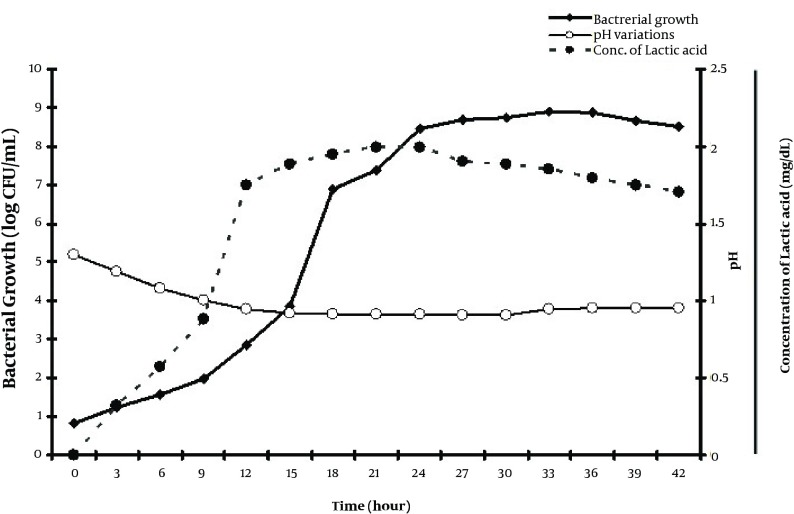
The Kinetics of Growth of *Lactobacillus casei* in MRS Media Incubated at 37˚C for 42 Hours Under Defined Microaerophillic Condition Using Anoxomat Incubator, the Variations in pH Values and Concentration of Lactic Acid. During Bacterial Generation

Figure 1 shows that *Lactobacillus casei *took approximately 9 hours to reach the log phase with generation time (TG) in 1 hour. This figure also displays the time course of lactic acid production and pH gradient of culture medium during generation of probiotics. The results showed formation of inhibition zone only around the wells which were contained lactic acid and fresh supernatant. Therefore, it was evidence that organic acids particularly lactic acid has the primary role in the antipathogenic effect of *Lactobacillus casei *supernatant than H_2_O_2_ and Bacteriocines. 

### 4.2. Comparison of Lactic Acid Concentration Between Different Times of Extraction

**Figure 2.**
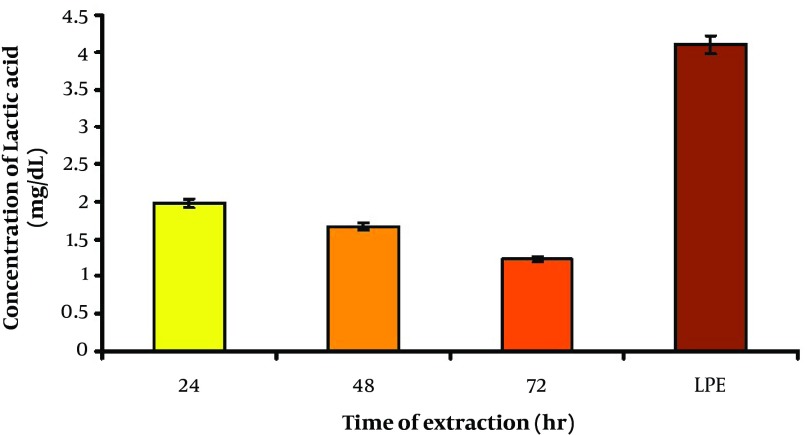
Comparison of Lactic Acid Concentration Between Samples of Different Time Extractions

Figure 2 shows that time of extraction caused a significant elevation in lactic acid concentration in 24 hours cultured group when compared to 48 and 72 hours cultured groups (P < 0.01). Its level significantly increased by lyophilization in LPE (P < 0.01) as compared to three other groups. 

### 4.3. Long Term Stability of Supernatant

**Figure 3.**
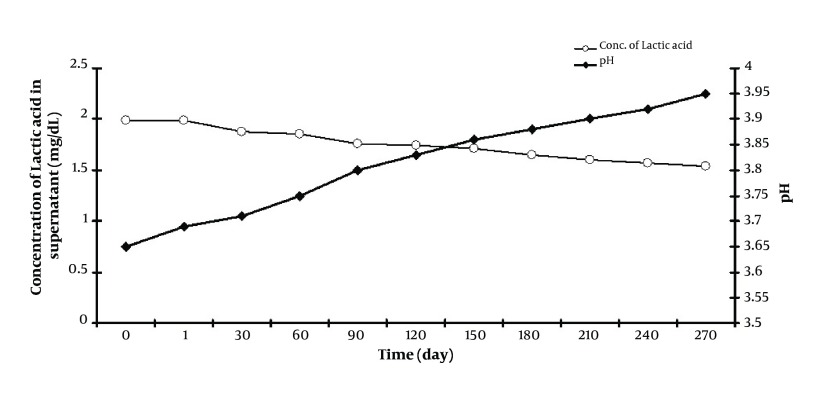
Long Term Stability of Supernatant of *L. casei* Stored at 4˚C for 9 Months. Values of pH and Lactic Acid Concentration in the Supernatant Were Determined During the Storage Period

Evaluation of pH values of acquired cell free supernatant showed a few elevations about 0.3-0.35 value during 9 months storage in 4˚C, but even these low changes could bias the stability of supernatant properties (Figure 3). On the contrary, the lactic acid levels of supernatant during this storage course reduced from 1.99 to 1.54 mg/dL. 

### 4.4. Antipathogenic Effect of Supernatant

**Figure 4.**
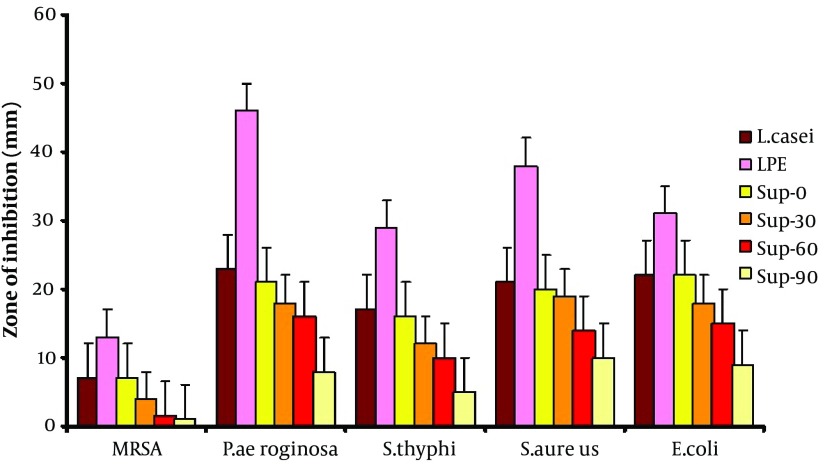
Comparison of Antibacterial Activity Between Different Samples of Probiotic Extract * MRSA: methicillin resistant *Staphylococcus aureus*

Figure 4 shows that, the antibacterial activity of LPE was significantly higher than that of other groups (P < 0.01). It also reveals similar antipathogenic effect of the fresh supernatant (Sup-0) with probiotics viable cells and there was a significant reduction in effectiveness of antibacterial properties of supernatant by time lapse as it was lowest in Sup-90. In all the samples, the greatest inhibition zone was toward *Pseudomonas aeruginosa, *and the narrowest zone was related to MRSA. 

### 4.5. Determination of MIC

MIC of Sup-0 and LPE has been tabulated in Table 1. It was found that MIC of LPE for *E. coli *and *S. typhi *is 0.062 mg/mL; whereas, for *P. aeruginosa *was 0.12 mg/mL, and 0.25 mg/mL for *S. aureus, *and MRSA was inhibited at 0.5 mg/mL. Besides, results also showed that Sup-0 could inhibit the *E. coli *, *P. aeruginosa *and *S. typhi *with 8 mg/mL; whereas, for *S. aureus *it was 16 mg/mL, and 32 mg/mL for MRSA. 

**Table 1.** Minimum Inhibitory Concentrations (MICs) of Fresh Supernatant (Sup-0) and LPE Against Several Pathogenic Strains

Indicator Pathogens	*E. coli*	*S. typhi*	*P. aeruginosa*	*S. aureus*	MRSA
**Concentration Range of Sup-0, mg/mL**					
4	+	+	+	+	+
8	-	-	-	+	+
16	-	-	-	-	+
32	-	-	-	-	-
64	-	-	-	-	-
**Concentration Range of LPE, mg/mL**					
0.031	+	+	+	+	+
0.062	-	-	+	+	+
0.12	-	-	-	+	+
0.25	-	-	-	-	+
0.5	-	-	-	-	-

### 4.6. Antioxidant Activity

**Figure 5.**
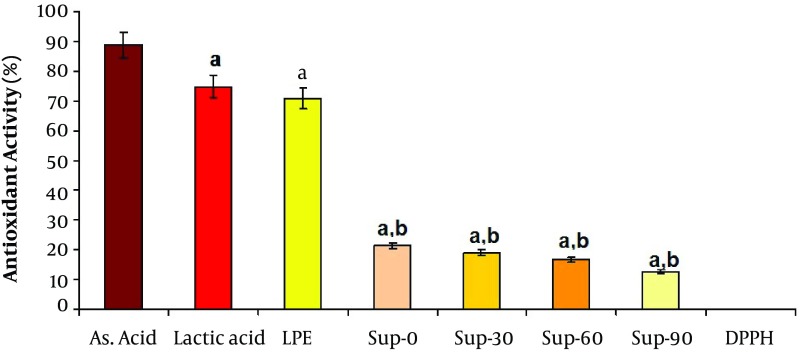
Comparison of Antioxidant Activity Between Different Samples of Probiotic Extract LPE: lyophilized probiotic extract, Sup-0: fresh probiotic extract, Sup-30: probiotic extract 30 days post extraction, Sup-60: probiotic extract 60 days post extraction, Sup-90: probiotic extract 90 days post extraction values are Mean ± SEM. a. Significantly different from Ascorbic acid at P < 0.01. b. Significantly different from Lactic acid at P < 0.01

Figure 5 confirms that all the five samples of probiotic supernatant exhibited potential antioxidant activity. LPE showed good antioxidant effect as compared to standard (Ascorbic acid), and could scavenge 70% DPPH free radical which was close to that of lactic acid (74.7%). Antioxidant activity represented significant reductive gradient in all samples of unlyophilized supernatant during storage time as it was at least in Sup-90. 

## 5. Discussion

This study demonstrated that the main beneficial effects of *L. casei* were related to their metabolites. LPE significantly had more antipathogenic effect, antioxidant activity, and stability in comparison to cell free supernatant. Concentration of the Lactic acid existing in the medium as the main metabolites product of the microflora, was chosen as a marker for evaluation of the activity of the *Lactobacillus*. Enzymatic lactate reagent kit due to its reliability, precision and reproducibility, was applied for detection of lactic acid in probiotic extracts (29, 30). The results from long term stability tests showed that, the stability of the amount of lactic acid in the supernatant was directly affected by the extraction time of cultured probiotic and also the time lapse of its storage. As it can be seen in the experiment that the time of extraction was 24 hours, the highest amount of lactic acid was observed contrary to the samples that were collected from culture medium at 48 and 72 hours intervals. Besides, pH variations of supernatant during storage were about 0.3-0.35 value for 9 months, and caused less acidic medium. This finding was in agreement with what was gained from assay of lactic acid during storage time. Thus, the main reason for pH-heightening of supernatant would decline in quantity of lactic acid and other organic acids. In spite of few changes in pH values and lactic acid quantity, it was not negligible because of its strong effect on the expected functions of extract. Hence, it was necessary to overcome this problem. To improve the stability of supernatant and also its concentrating, freeze-drying was applied as a suitable method. During freeze-drying, dehydration was occurred without exposing the obtained bioextract (or supernatant) to high temperatures which lead to preservation of its structure (31, 32). As results showed, lactic acid concentration was higher in lyophilized probiotic extract (LPE) when compared to normal supernatant which caused more antipathogenic effects. The data of MICs was evident that LPE is active against both Gram positive and negative bacteria, but more effective against Gram negatives at low concentration. Although there are different methods for measuring antioxidant activity, the DPPH method as standard one was used. Its results followed similar manner with antipathogenic effects of LPE and confirmed its potency contrary to normal supernatant. To ensure the safety of LPE, its toxicity was investigated in vivo using health murine model where any of animals in test-group had received high quantity of LPE. Fortunately, no significant toxicity or even unexpected adverse effects or unusual activities was observed in animals (33). According to findings from the in vitro and in vivo studies, it is apparent that the main mechanism of *Lactobacillus* correlated with its antimicrobial and antioxidant activity is the pH-lowering effect caused by the production of organic acids and boosting host immunity (14, 16, 34). Using lyophilized probiotic extract can be attended as an innovation in strategy of probiotic products preparation. Whereas in last decade, probiotic-containing-products have been added intensively in food products mainly dairy-products, dietary and medicinal supplements, and more recently, therapeutic and prophylactic medicines, this finding would be a practical substitute for using probiotic live cells.

In summary, as a novel technology, lyophilized cell-free probiotic extract or LPE, displayed effective bactericidal and antioxidant activities without any toxic effects even at high quantity. Although, this approach requires further investigations, it gives new interest concept of probiotic-therapy as well as replacement for probiotic live cells, and also good antioxidant ingredient in nutritional and medicinal industries.
